# Parameterized quantum circuits as universal generative models for continuous multivariate distributions

**DOI:** 10.1038/s41534-025-01064-3

**Published:** 2025-07-22

**Authors:** Alice Barthe, Michele Grossi, Sofia Vallecorsa, Jordi Tura, Vedran Dunjko

**Affiliations:** 1 Quantum Technology Initiative, CERN, Geneva, Switzerland; 2 Applied Quantum Algorithms, Universiteit Leiden, Leiden, the Netherlands; 3 Instituut-Lorentz, Universiteit Leiden, Leiden, the Netherlands

**Keywords:** Quantum information, Qubits, Computer science

## Abstract

Parameterized quantum circuits are a key component of quantum machine learning models for regression, classification, and generative tasks. Quantum Circuit Born machines produce discrete distributions over bitstrings whose length is exactly the number of qubits. To allow for distributions on continuous variables, new models have been introduced where classical randomness is uploaded into quantum circuits and expectation values are returned with a dimensionality decoupled from qubit number. While these models have been explored experimentally, their expressivity remains underexplored. In this work, we formalize this family and establish its theoretical foundation. We prove the universality of several variational circuit architectures for generating continuous multivariate distributions and derive tight resource bounds to reach universality using tools related to the Holevo bound. Our results reveal a trade-off between the number of qubits and measurements. We further explore relaxed notions of universality and present a practical use case, outlining potential domains for quantum advantage.

## Introduction

Parameterized quantum circuits are the centerpiece of numerous approaches to machine learning on quantum computers, motivated by several near-term hardware limitations^1,2^. The use of these models for supervised learning has been widely studied, for example, in solving regression problems^3^ where the goal is to assign continuous labels to data points. Variational algorithms have also been explored in so-called generative modeling tasks, where the objective is to generate new samples following a distribution that generated the training data^4^.

A prominent example is the quantum Boltzmann machine (QBM) as introduced in ref. ^5^, modeling distributions with discrete support, in which data is represented as the thermal state of an Ising model. In^6^ the training based on the relative entropy of QBM for more general models is investigated, and it is shown that the training cannot be simulated with classical computers unless BPP = BQP. The QBM has been compared in-extenso in^7^ with another quantum generative model for discrete variables, the quantum circuit born machines (QCBM). In QCBM^8^ a probability distribution over *n*-bit strings is stored in a *n*-qubit pure state. They may be trained to minimize the maximum mean discrepancy but also as generators in generative adversarial networks (GANs) settings^9–12^, yielding the so-called Qu-GAN.

Going beyond distributions with discrete support, an approach to model distributions where the random variable can in principle take any value within a continuous interval has been introduced in ref. ^13^. In such models, the quantum circuit takes classical randomness as input and outputs expectation values, consequently, we call this model *expectation value samplers*. This model has been used as a quantum generator in the context of quantum generative adversarial networks (GAN), with applications including image generation^14,15^, high energy physics^16^, and chemistry^17^. In all these applications, the training is performed in the GAN setting.

While for QCBMs, the expressivity and universality have been clarified^8,18^, in contrast, expectation value sampling models are not so well understood. In particular, an interesting feature of expectation value sampling is that the dimension of the output is not inherently tied to the number of qubits used. In this work, we focus on the expressivity of the generators based on expectation value sampling depending on the number of qubits and the observable norm. In light of the numerous works^14–17,19–23^ that successfully used expectation value samplers, this work aims to solidify the theoretical ground that supports them by formally analyzing their expressivity. The formal analysis of the trainability of such models, while being a matter of critical importance, is not in the scope of this work.

The focus of this work is to show that parameterized quantum circuits are universal generative models and precisely characterize their expressivity. Our first main result is the existence of two universal families of expectation value samplers. We probe tight bounds relating to resource limitations, that is, necessary conditions on resources for expectation value sampling models to be universal. Specifically, we show that reaching universality for very high-dimensional distributions requires either a very large number of qubits or a very large number of measurements. This may serve as a backbone for future fine-grained resource cost analyses. As additional tools to analyze expressivity, we discuss choices of random variables, circuit encoding, and observables, which we hope will guide future designs. Finally, we motivate the use of expectation value samplers with respect to other existing quantum generative models by providing a natural sampling task for these models.

## Results

In this section, we introduce expectation value sampling models and define precisely the universality property for generative models. We state formally our first main result, namely the existence of two universal families of expectation value samplers. We then present our second main result, the minimum resource requirements for expectation value samplers to be universal.

### Expectation value sampling

**Fig. 1:**
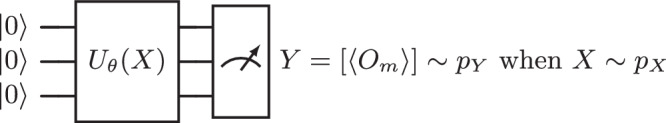
Expectation value sampling model: a random vector is classically sampled. It is used to generate a random quantum state using a parameterized quantum circuit. The expectation values of fixed observables are returned as the output random vector.

The expectation value sampling procedure goes as follows. A random variable is classically sampled and used as input to a parameterized quantum circuit, which specifies a random quantum state. The expectation values of fixed observables are measured and returned as another random variable. We illustrate this procedure in Fig. 1, and provide a formal definition below.

#### Definition 1

(Expectation value sampling model). An expectation value sampling model on *n* qubits is defined by (*U*_*θ*_, **O**, *p*_*X*_), where ${U}_{\theta }:{\mathcal{X}}\subseteq {{\mathbb{R}}}^{L}\to {\mathcal{U}}({2}^{n})$ is a parameterized quantum circuit taking data as input and returning a matrix in the 2^*n*^-dimensional unitary group ${\mathcal{U}}({2}^{n})$, ${\bf{O}}={({O}_{m})}_{1\le m\le M}$ is a vector of *M* observables, and ${p}_{X}:{\mathcal{X}}\subseteq {{\mathbb{R}}}^{L}\to {\mathbb{R}}$ is the input distribution. We define the associated mapping *f* as follows:

1 $$x\in {\mathcal{X}}\mathop{\to }\limits^{f}{\left(\left\langle 0\right\vert {U}_{\theta }{(x)}^{\dagger }{O}_{m}{U}_{\theta }(x)\left\vert 0\right\rangle \right)}_{1\le m\le M}.$$

The output of the model is a sample drawn from the distribution *p*_*Y*_ with *Y* = *f*(*X*) ~*p*_*Y*_ when *X* ~*p*_*X*_.

It is important to note that, in contrast to the quantum circuit Born machine, the randomness does not come from the measurement process, but from the classical randomness provided as an input to the quantum circuit. Another difference is that expectation value samplers have continuous support (absolutely continuous random variables, see Supplementary Information). The central question of this work is whether such a model can generate any multivariate distribution, more precisely, whether expectation value sampling is a universal generative model, according to the definition we will give in the next subsection.

### Universal generative model family

In this subsection, we precisely define universal generative model families. We choose the Wasserstein distance to quantify the closeness of two distributions. For reasons we detail in the Supplementary Information. While the Wasserstein distance is impractical for training purposes, it is still a relevant metric in the analysis of the expressivity. A main reason is that it naturally arises as the mathematical concepts we use in this work are related to optimal transport. Other common losses are the Kullback–Leibler divergence, which is not well suited to this work as it is not symmetric and not a true metric. Another frequently used loss is the maximum mean discrepancy, which is easier to compute, but relative to a chosen kernel, and therefore not an absolute property of the closeness between two distributions.

#### Definition 2

(Universal generative model family) A generative model is a family of parameterized sampling procedures that enable sampling from a corresponding set of *M*-dimensional probability density functions ${\mathcal{P}}({\mathcal{X}})$ on ${\mathcal{X}}\subseteq {{\mathbb{R}}}^{M}$.

A generative model is called universal if for every probability density function *q* on ${\mathcal{X}}$ there exists a sequence ${\{{p}_{k}| {p}_{k}\in {\mathcal{P}}({\mathcal{X}})\}}_{1\le k\le \infty }$ such that it converges to *q* in the Wasserstein distance *W*.

2 $$W(q,{p}_{k})\mathop{\longrightarrow }\limits_{k\to \infty }0$$

Importantly, universality is defined on a given support noted as ${\mathcal{X}}$ in Definition 2. For this work, we choose the support as the hypercube ${\mathcal{X}}={[-1,1]}^{M}$, because the first step of most machine learning pipelines is to rescale the data to fit on a given interval, usually [−1, 1]. Notably, choosing universality on a cubic support [−1, 1]^*M*^ allows the expression of fully independent variables. Restricting ${\mathcal{X}}$ to smaller subsets would yield constraints on the dependence relationships expressible. For example, proving the universality of a family of models for Dirichlet distributions would correspond to choosing ${\mathcal{X}}$ in the hyperplane where all variables sum up to one and are positive.

### Two families of expectation value samplers as universal generators

First, building on work by Pérez-Salinas et al.^24^, we show that *n*-qubit expectation value samplers with constant observable norm are universal for *M*-dimensional distributions with constant support radius, for *M* = *n*.

#### Theorem 1

For any M, for all *M*-dimensional probability density functions *p*_*Z*_ with support included in [−1, 1]^*M*^, and for all accuracy *ϵ* > 0 there exists a *M*-qubit circuit *U* and set of *M* observables **O** with unit spectral norm ∥*O*_*m*_∥ = 1 such that the expectation value sampling model (*U*, **O**, *p*_*X*_) where *p*_*X*_ is the uniform distribution on [0, 1]^*M*^ yields a probability density function *p*_*Y*_ that is *ϵ*-close to *p*_*Z*_ in the Wasserstein distance.

**Fig. 2:**
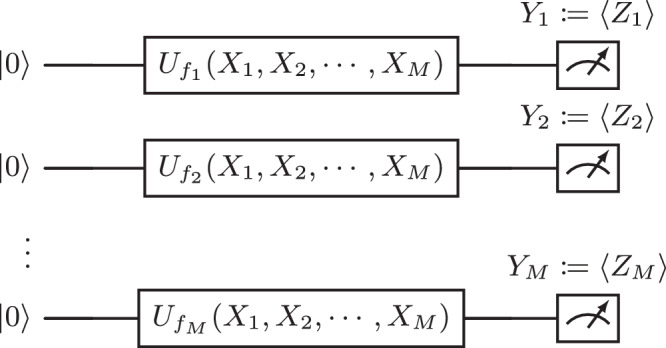
*Product Encoding* Circuit as a universal generator yielding the random variable *Y* = *g*(*X*) when *X* ~*U*([0, 1]^*M*^), by stacking circuits from^24^*U*_*f*_ approximating *f*, and defining *f*_*m*_:= (*g*_*m*_ + 1)/2.

**Fig. 3:**
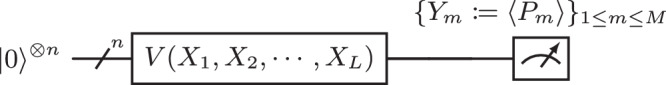
*Observable Dense Encoding* Circuit as a universal generator, based on a universal state preparation circuit *V*, with each parameterized gate replaced by a circuit from^24^. $n=\log (M+1)$ and ${P}_{m}=2M\left\vert m\right\rangle \left\langle m\right\vert -I$.

We derive an explicit construction, illustrated in Fig. 2, for this circuit, which yields product states; hence, we name it “*product encoding*”. It uses the same number of qubits as the dimension of the output distribution. This construction defeats one advantage of expectation value samplers, that is, the dimension of the output not being directly linked to the number of qubits used. This raises the question of the existence of a more qubit-frugal family of universal circuits, in which the output dimension is (much) larger than the qubit number. We show the existence of such a family, at the cost of allowing for observables to have large norms. We formalize this in the following theorem and illustrate the explicit construction of the circuit in Fig. 3, and call it “*observable-dense encoding*” expectation value sampler.

#### Theorem 2

For any *M*, for all *M*-dimensional probability density functions *p*_*Z*_ with support included in [−1, 1]^*M*^, and for all accuracy *ϵ* > 0 there exists a $n=\Theta (\log M)$-qubit circuit *U* taking *L* input variables and set of *M* observables **O** with spectral norm ∥*O*_*m*_∥ ∈ Θ(*M*) such that the expectation value sampling model (*U*, **O**, *p*_*X*_) where *p*_*X*_ is the uniform distribution on [0, 1]^*L*^ yields a probability density *p*_*Y*_ that is *ϵ*-close to *p*_*Z*_ in the Wasserstein distance.

In this subsection, we have provided *sufficient* conditions for expectation value samplers to be universal. In particular, we have shown the existence of two extremal families of parameterized quantum circuits that are universal generators on [−1, 1]^*M*^. There is a *product encoding* design, illustrated in Fig. 2, with *n* = *M* qubits and with unit norm observables (local Pauli), and an *observable-dense encoding* design, illustrated in Fig. 3, with $n=\log (M)$ qubits and *M* norm observables (amplified probabilities of bitstrings). While one has a large number of qubits for a constant observable norm, the other has a logarithmic number of qubits but large observable norms. This hints that there might be a trade-off between the number of measurements and the number of qubits necessary to achieve universality. In the next section, we prove this is the case by proving some *necessary* universality conditions.

### Necessary conditions for universality

In this subsection, we prove two necessary conditions on resources for expectation value samplers to be universal. As previously mentioned, an appealing feature of expectation value sampling models is that the dimension of the output vector is a priori independent of the number of qubits *n*, unlike in the case of quantum Born machines, where each qubit corresponds to exactly one binary random variable. In particular, we can imagine using just a single qubit with an arbitrary number of observables *O*_*m*_ to generate an *M*-dimensional random vector. However, it is obvious that in this case, the random variables corresponding to each observable cannot all be fully independent. Indeed, any observable can be expressed as a linear combination of the three Pauli matrices. Therefore, the distribution output by a single qubit expectation value sampler will have at most three degrees of independence, and for *M* > 3 it is impossible to reach universality because it is impossible to approximate, e.g., the 4-dimensional uniform distribution. Extending this reasoning to several qubits, we find the first necessary condition, that the dimension of the target dimension has to be lower than or equal to the dimension of the space of observables. We formalize this in the following lemma.

#### Lemma 1

For an *n*-qubit expectation value sampling model (*U*_*θ*_(*x*), **O**, *p*_*X*_) to be able to approximate any distribution with support in [−1, 1]^*M*^ to any accuracy *ϵ* > 0, it is necessary that *M* ≤ 4^*n*^ − 1.

The second necessary condition for an expectation value sampling model to be universal on distributions with support in [−1, 1]^*M*^ can be derived using a combination of Holevo’s bound found in ref. ^25^ and Chernoff bound. We formalize it in the following theorem.

#### Theorem 3

For an *n*-qubit expectation value sampling model (*U*_*θ*_, **O**, *p*_*X*_) to be able to approximate any distribution with support in [−1, 1]^*M*^ to any accuracy *ϵ* > 0 with respect to the Wasserstein distance, it is necessary that for every *m* ≤ *M*, both equations are satisfied:

3 $${\lambda }_{\min }({O}_{m})\le -1+\epsilon \,{\text{and}}\,{\lambda }_{\max }({O}_{m})\ge +1-\epsilon \,,$$

4 $$n\in \Omega \left(\frac{M{(1-\epsilon )}^{2}}{\parallel {O}_{m}{\parallel }^{2}}\right)\,.$$

with ${\lambda }_{\min /\max }(O)$ returning respectively the minimum and maximum eigenvalues of observable *O*.

**Fig. 4:**
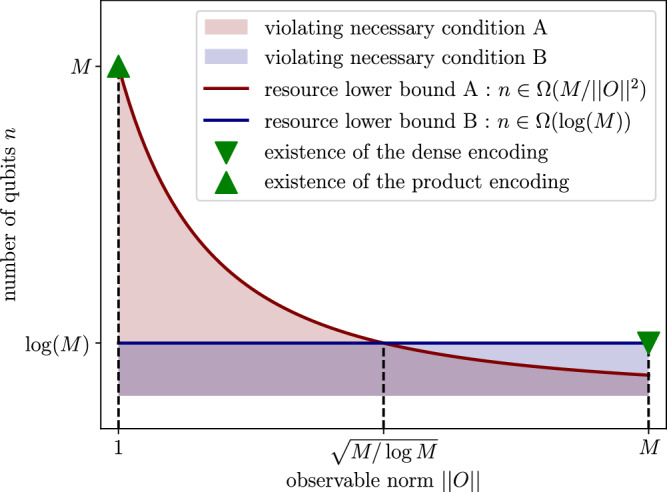
Visual summary of results. We show the asymptotic use of resources for expectation value samplers to reach universality for an *M*-dimensional target distribution: the necessary conditions, as well as the existence of families as in Figs. 2 and 3.

The combination of both previous necessary conditions is the second central result of this paper. It formalizes that even if expectation value sampling models may output arbitrarily large dimensional distributions, in practice, their expressivity is limited by the number of qubits and observables. It is a natural question to ask whether the universal families we found previously satisfy the above necessary conditions. We illustrate such considerations in Fig. 4. For target distributions with exponentially large dimensionality, the two universal families are (almost) asymptotically optimal.

We may conjecture that there exists a family of (Pareto) optimal circuits, balancing between observable norms and qubit numbers, with varying *encoding densities* in between the two extremal ones. In practice, the observable spectral norm relates to the number of measurements required to approximate it up to an additive accuracy. This highlights a trade-off between the number of qubits and the number of measurements, or space versus time complexity, which we formalize in the next subsection.

### Trade-off: qubits vs measurements

To estimate an expectation value up to a desired constant additive error, the number of measurements required is proportional to the norm of the observables. Therefore, large observable norms require a large number of measurements.

#### Lemma 2

(informal) To guarantee that the *M*-dimensional distribution coming from an expectation value sampler performing *T* measurements is *ϵ*-close in the Wasserstein distance to the ideal shot-noise-free distribution, it is sufficient that

5 $$T\in \Theta \left(\frac{M\!\!\parallel\!\! O\!\parallel }{{\epsilon }^{2}}\right).$$

Therefore, the necessary conditions we derived previously ultimately highlight a trade-off between the number of qubits and the number of measurements. We show that to be universal for very high-dimensional distributions ${\mathcal{X}}$, expectation value samplers need either a very large number of qubits or a very large number of measurements. This is in contrast with the initial intuition that expectation value samplers may generate high-dimensional distributions independently of the number of qubits.

## Discussions

The arguments we present to derive resource lower bounds necessary for achieving universality in expectation value samplers (EVS) are driven by the number of independent variables of the target distribution. Because our definition of universality allows for full independence relationships across all dimensions, the number of independent variables is equal to the dimension of the target distribution. A more refined perspective emerges when considering families of distributions with inherent dependencies and allows for more economical sampling strategies than the ones requiring full independence, as presented in Fig. 4. Two concrete examples serve to emphasize this point. First, consider the family of distributions that are *M*-dimensional but have their support confined within a 2-dimensional linear subspace. In this case, using the expectation value sampler described in ref. ^24^ with the observables $\sqrt{2}X,\sqrt{2}Z$, we achieve a universal generator on a single qubit for this 2-linear family, independent of the target distribution dimension *M*. This economy of representation underscores the capacity of EVS to exploit structural sparsity effectively. Next, consider *M*-dimensional Dirichlet distributions, characterized by non-negative coordinates summing to unity. Here, the observable dense encoding circuit, configured with $n=\log M$ qubits, enables universality with the raw probabilities as observables (Fig. 3). This model achieves universal representation with exponentially fewer qubits compared to the general setting that allows for full independence, leveraging that the Born rule naturally gives rise to Dirichlet distributions. These findings suggest that EVS models are particularly well-suited for generating highly correlated distributions. The Dirichlet case is illustrative, as its normalization condition aligns seamlessly with that of quantum states, lending itself naturally to the expectation value sampling paradigm.

Building on this, we discuss the impact of design choices in EVS, specifically the selection of observables, the encoding *U*(*x*), and the input distribution *p*_*X*_, all of which are detailed further in the Supplementary Information. The choice of observables directly influences the subspace of the distribution spanned by the EVS. Furthermore, regarding the encoding structure *U*(*x*), we realize that, analogously to the way parameterized quantum circuits may have an exact finite Fourier decomposition, expectation value samplers may have an exact finite Generalized Polynomial Chaos Expansion. In this context, the choice of the uniform distribution as an input distribution arises naturally, while in contrast to classical GANs, a Gaussian distribution yields undesirable inductive bias. Thanks to this proximity between the supervised and the generative context, results on the expressivity of quantum reuploading models^26^ transfer naturally to the encoding circuits of expectation value samplers. In addition, as the Expectation Value samplers effectively learn a function in full analogy to how it is done in supervised learning, it is clear that much of the current discussion on the trainability of parametrized circuits for supervised learning, and of more general variational algorithms, will apply to the Expectation Value samplers. It will have the same bottlenecks of overly expressive architectures^27^, but also the various classes of new techniques that mitigate these by designs that balance trainability and dequantizability^28–30^, and other more practical methods which mitigate trainability problems^31^.

While we characterized important properties of expectation value samplers, we did not address the question of whether it is a good idea to use them. Expectation value samplers cannot be proven to be a path for certain types of quantum advantage as directly as is the case in Quantum Circuit Born Machines. QCBM is suited for quantum use cases by design as they rely on the Born rule for generation of samples, and so correspond to distributions obtained by a measurement of a genuinely quantum state. In contrast, expectation value samplers rely on classical randomness and, rather than requiring sampling from the full distribution of quantum measurements, are defined around expectation values only. Thus, the hardness of simulating distributions from expectation value samplers does not connect straightforwardly to any hardness-of-sampling results established in the domain of quantum supremacy results^18^. Nonetheless, expectation value samplers still consist of genuinely quantum computations, and arguments for non-simulatability can be made. Assuming BQP is not in BPP, there exists no polynomial time algorithm that takes a classical description of an arbitrary expectation value sampler *A* as input and outputs a sample from a distribution that is epsilon close to that of the output of *A*. Take, as an example, the case where a hard-to-simulate circuit does not depend on input data. This yields a Dirac delta distribution for which there exist classical samplers to efficiently sample from it, however, such classical samplers cannot be easily found based on the classical description of the expectation value sampler. It may be possible to construct stronger advantage arguments where we find the existence of an expectation value sampler such that its output distribution cannot be sampled by any polynomial-time randomized Turing machine (subject to standard assumptions). This raises the question: Do expectation value samplers also correspond to any natural quantum task?

A natural domain of application for EVS emerges in many-body physics, particularly in the study of disordered systems. We propose the example of spin glasses, which with interaction strengths *J*_*i*,*j*_ taken at random, are governed by the Hamiltonian:

6 $$H(J)=\sum _{\langle i,j\rangle }{J}_{i,j}{S}_{i}{S}_{j}-M\sum _{i}{S}_{i}\,.$$

**Fig. 5:**
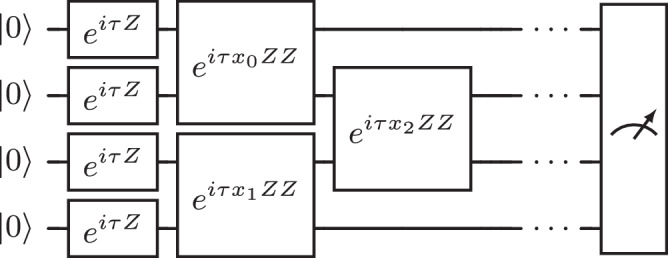
Expectation value sampler as the Trotterization of the spin glass Hamiltonian in (6).

In this model, the interaction strengths *J*_*i*,*j*_ are taken at random. Consider the quench dynamics of such a system, initialized as the ground state of the Hamiltonian without any interaction *H*(0), that is the zero state. The interactions are instantly turned on for a fixed time, and a set of interactions is taken at random. Then one may compute the expectation value of a set of observables of interest, such as local spin or local correlators, measured using several copies of the same realization of interaction strengths. This data is inherently quantum, and the Trotterization of this time evolution constitutes a solution in the form of an expectation value sampler where the output can be made arbitrarily close to the target distribution. We exemplify such a circuit for a chain geometry on four qubits in Fig. 5. Using two registers with different evolution times, one could even extract a two-time correlation function $C(t,t^{\prime} )={\sum }_{i}\langle {S}_{i}(t){S}_{i}(t^{\prime} )\rangle$, which in turn gives precious information about the Edwards–Anderson parameter, an order parameter for spin glasses.

## Methods

The proofs of all theorems are available in the Supplementary Information, but we provide high-level ideas in this section. First, we highlight a core concept in this work, variable transformation, and how we use it to prove universality. Subsequently, we explain the main steps of the proof and construction of the two universal families as a high-level summary of Section II of the SI.

### Random variable transformation

A core concept in this work is that of random variable transformation. In this subsection, we introduce it and provide some of the associated fundamental properties.

The first step in the analysis of expectation value samplers is the observation that they are processes that map an input random vector (parameterizing the quantum circuit) to an output random vector (the expectation values of a set of observables). The literature on optimal transport and measure theory^32^ states that for every pair of absolutely continuous random variables of the same dimension, there exists a mapping to transform one into the other.

#### Lemma 3

For every pair of absolutely continuous probability density functions ${p}_{X}\in {\mathcal{P}}({\mathcal{X}}\subseteq {{\mathbb{R}}}^{M})$ and ${p}_{Y}\in {\mathcal{P}}({\mathcal{Y}}\subseteq {{\mathbb{R}}}^{M})$, there exists a mapping $f:{\mathcal{X}}\to {\mathcal{Y}}$, that maps *Y* ~ *p*_*Y*_ to *X* ~ *p*_*X*_ as *Y* = *f*(*X*).

We give intuition on how to construct this mapping to be bounded piece-wise continuous in the Supplementary information. Many generative modeling systems, including Generative Adversarial Networks, Variational Auto-Encoders, and normalizing flows, rely on this idea to generate arbitrary distributions. In particular, the initial distribution is chosen to be simple, and then by altering the mapping applied to this random input, we obtain a rich spectrum of possible output distributions.

Then, sufficient conditions for a family of mappings to yield a universal generative model, in the sense of Definition 2, are well-known and expressed in terms of the universality of mappings themselves. From ref. ^33^, it is sufficient for a family of mappings to be dense in the set of all monotonically increasing functions in the pointwise convergence topology to yield a universal generative model. We explain the difference between pointwise topology and uniform topology in the Supplementary Information. Since these are sufficient conditions and monotonic functions are included in all functions, the following holds.

#### Lemma 4^33^

If a family of mappings ${\mathcal{G}}=\{g:{\mathcal{X}}\subseteq {{\mathbb{R}}}^{M}\to {\mathcal{Y}}\subseteq {{\mathbb{R}}}^{M}\}$ is dense in the set of all functions in the pointwise convergence topology, then this family of mappings ${\mathcal{G}}$ together with a probability density function *p*_*X*_ with non-zero support on ${\mathcal{X}}$ yields a universal generative model family on ${\mathcal{Y}}$ (cf Definition 2).

In classical machine learning, such a notion of universality is common. It has been proven for several families of mappings in the context of normalizing flows, which are mappings with the additional property of being invertible: generic triangular mappings^32^, neural network mappings^34^, and polynomial mappings^35^.

In the next section, to prove the universality of expectation value samplers, we make explicit the connection between universal mapping families and the known results on the universality of parameterized quantum circuits as supervised learning models.

### Universality proofs

In recent literature, several universality properties of parameterized quantum circuits have been proven. In^24^, a family of single-qubit quantum circuits with an increasing number of layers is proven to be universal in the uniform sense for continuous multidimensional input functions as complex coordinates of the quantum state in the computational basis.

We extend this existing result to fit our needs, as follows. First, we modify universality results from functions as coordinates in the computational basis to functions as the expectation value of an observable. Then we broaden the universality of quantum reuploading models to some discontinuous functions by relaxing the required strength of convergence. More precisely, we go from the uniform density in bounded continuous functions to the pointwise density in bounded piecewise continuous functions. Finally, by stacking *M* universal circuits, we extend universality to multivariate output functions. All these extensions of ref. ^24^ together yield the theorem below.

#### Theorem 4

For every natural number *M*, for every mapping $f\in {\mathcal{B}}({[0,1]}^{M}\to {[-1,1]}^{M})$, there exists a sequence of sets of *M* single-qubit quantum circuits and unit norm observables (indexed by *k*).

7 $${\left\{{({U}_{k,m}:{[0,1]}^{M}\to {\mathcal{U}}(2),{O}_{k,m})}_{1\le m\le M}\right\}}_{1\le k\le \infty }$$

such that the sequence of functions ${\{{g}_{k}\}}_{1\le k\le \infty }$ defined as

8 $${g}_{k,m}(x)=\left\langle 0\right\vert {U}_{k,m}{(x)}^{\dagger }{O}_{k,m}{U}_{k,m}(x)\left\vert 0\right\rangle$$

converges pointwise to *f*, where ${\mathcal{B}}$ is the set of piecewise continuous functions, and the norm of the observable is the spectral norm.

The theorem above shows that there exists a family of *M*-qubit circuits with unit norm observables that yield a family of functions that is pointwise dense in the set of bounded piece-wise continuous functions. This matches the sufficient conditions of Lemma 4 for mappings to yield a universal generative model. This yields that there exists a family of expectation value sampling models as defined in Definition 1 and illustrated in Fig. 2 that is universal in the sense of Definition 2.

For the *observable dense encoding* we follow a similar strategy, but instead, each output variable is encoded as the overlap between the output state and each computational basis state. With the normalization of the quantum states, the observables are projectors on computational basis states, amplified by a factor proportional to the dimension of the target distribution. More details are provided in the Supplementary Information.

## Supplementary information

Supplementary Information pdf

## Data Availability

No datasets were generated or analysed during the current study.

## References

[1] Bharti, K. et al. Noisy intermediate-scale quantum algorithms. *Rev. Mod. Phys.* **94**, 015004 (2022).

[2] Cerezo, M. et al. Variational quantum algorithms. *Nat. Rev. Phys.* **3**, 625–644 (2021).

[3] Macaluso, A., Clissa, L., Lodi, S. & Sartori, C. A Variational algorithm for quantum neural networks. In: (eds Krzhizhanovskaya, V. V. et al.) *Computational Science—ICCS 2020*, Lecture Notes in Computer Science, pp 591–604 (Springer International Publishing, Cham, 2020).

[4] Tian, J. et al. Recent advances for quantum neural networks in generative learning. *IEEE Trans. Pattern Anal. Mach. Intell.* **45**, 12321–12340 (2023). 37126624 10.1109/TPAMI.2023.3272029

[5] Amin, M. H., Andriyash, E., Rolfe, J., Kulchytskyy, B. & Melko, R. Quantum Boltzmann machine. *Phys. Rev. X* **8**, 021050 (2018).

[6] Kieferová, M. & Wiebe, N. Tomography and generative training with quantum Boltzmann machines. *Phys. Rev. A* **96**, 062327 (2017).

[7] Cheng, S., Chen, J. & Wang, L. Information perspective to probabilistic modeling: Boltzmann machines versus born machines. *Entropy* **20**, 583 (2018). 33265672 10.3390/e20080583 PMC7513111

[8] Liu, J.-G. & Wang, L. Differentiable learning of quantum circuit Born machines. *Phys. Rev. A* **98**, 062324 (2018).

[9] Lloyd, S. & Weedbrook, C. Quantum generative adversarial learning. *Phys. Rev. Lett.* **121**, 040502 (2018). 30095952 10.1103/PhysRevLett.121.040502

[10] Dallaire-Demers, P.-L. & Killoran, N. Quantum generative adversarial networks. *Phys. Rev. A* **98**, 012324 (2018).

[11] Zeng, J., Wu, Y., Liu, J.-G., Wang, L. & Hu, J. Learning and inference on generative adversarial quantum circuits. *Phys. Rev. A* **99**, 052306 (2019).

[12] Zoufal, C., Lucchi, A. & Woerner, S. Quantum generative adversarial networks for learning and loading random distributions. *npj Quantum Inf.* **5**, 1–9 (2019).

[13] Romero, J. & Aspuru-Guzik, A. Variational quantum generators: generative adversarial quantum machine learning for continuous distributions. *Adv. Quantum Technol.* **4**, 2000003 (2021).

[14] Huang, H.-L. et al. Experimental quantum generative adversarial networks for image generation. *Phys. Rev. Appl.* **16**, 024051 (2021).

[15] Chang, S. Y., Thanasilp, S., Saux, B. L., Vallecorsa, S. & Grossi, M. Latent style-based quantum GAN for high-quality image generation. Preprint at *arXiv* http://arxiv.org/abs/2406.02668 (2024).

[16] Bravo-Prieto, C. et al. Style-based quantum generative adversarial networks for Monte Carlo events. *Quantum* **6**, 777 (2022).

[17] Kao, P.-Y. et al. Exploring the advantages of quantum generative adversarial networks in generative chemistry. *J. Chem. Inf. Model.* **63**, 3307–3318 (2023). 37171372 10.1021/acs.jcim.3c00562 PMC10268960

[18] Coyle, B., Mills, D., Danos, V. & Kashefi, E. The Born supremacy: quantum advantage and training of an Ising Born machine. *npj Quantum Inf.* **6**, 1–11 (2020).

[19] Tsang, S. L., West, M. T., Erfani, S. M. & Usman, M. Hybrid quantum-classical generative adversarial network for high resolution image generation. *IEEE Trans. Quantum Eng.* **4**, 1–19 (2023).

[20] Chakrabarti, S., Yiming, H., Li, T., Feizi, S. & Wu, X. Quantum Wasserstein generative adversarial networks. In: (eds Wallach, H. et al.) *Advances in Neural Information Processing Systems*, vol. 32 https://proceedings.neurips.cc/paper_files/paper/2019/file/f35fd567065af297ae65b621e0a21ae9-Paper.pdf. (Curran Associates, Inc., 2019).

[21] Qu, Z., Chen, W. & Tiwari, P. HQ-DCGAN: Hybrid quantum deep convolutional generative adversarial network approach for ECG generation. *Know. Based Syst*. 10.1016/j.knosys.2024.112260 (2024).

[22] Shu, R., Xu, X., Yung, M.-H. & Cui, W. Variational quantum circuits enhanced generative adversarial network. Preprint at *arXiv* http://arxiv.org/abs/2402.01791 (2024).

[23] Silver, D. et al. MosaiQ: quantum generative adversarial networks for image generation on NISQ computers. Preprint at *arXiv* http://arxiv.org/abs/2308.11096 (2023).

[24] Pérez-Salinas, A., López-Núñez, D., García-Sáez, A., Forn-Díaz, P. & Latorre, J. I. One qubit as a Universal Approximant. *Phys. Rev. A* **104**, 012405 (2021).

[25] Ambainis, A., Nayak, A., Ta-Shma, A. & Vazirani, U. Dense quantum coding and quantum finite automata. *J. ACM* **49**, 496–511 (2002).

[26] Barthe, A. & Pérez-Salinas, A. Gradients and frequency profiles of quantum re-uploading models. Preprint at *arXiv* http://arxiv.org/abs/2311.10822 (2023).

[27] Cerezo, M. et al. Does provable absence of barren plateaus imply classical simulability? Or, why we need to rethink variational quantum computing. Preprint at *arXiv* http://arxiv.org/abs/2312.09121 (2024). 10.1038/s41467-025-63099-6 PMC12378457 40855050

[28] Gyurik, C. & Dunjko, V. Exponential separations between classical and quantum learners. Preprint at *arXiv* http://arxiv.org/abs/2306.16028 (2023).

[29] Gil-Fuster, E., Gyurik, C., Pérez-Salinas, A. & Dunjko, V. On the relation between trainability and dequantization of variational quantum learning models. Preprint at *arXiv* http://arxiv.org/abs/2406.07072 (2024).

[30] Molteni, R., Gyurik, C. & Dunjko, V. Exponential quantum advantages in learning quantum observables from classical data. Preprint at *arXiv* http://arxiv.org/abs/2405.02027 (2024). 10.1038/s41534-025-01162-2 PMC12846916 41613165

[31] Mhiri, H. et al. A unifying account of warm start guarantees for patches of quantum landscapes. Preprint at *arXiv*10.48550/arXiv.2502.07889 (2025).

[32] Bogachev, V. I., Kolesnikov, A. V. & Medvedev, K. V. Triangular transformations of measures. *Sbornik* **196**, 309 (2005).

[33] Kobyzev, I., Prince, S. D. & Brubaker, M. A. Normalizing flows: an introduction and review of current methods. *IEEE Trans. Pattern Anal.* **43**, 3964–3979 (2021). 10.1109/TPAMI.2020.2992934 32396070

[34] Huang, C.-W., Krueger, D., Lacoste, A. & Courville, A. Neural autoregressive flows. In: *Proc. 35th International Conference on Machine Learning*, pp. 2078–2087. https://proceedings.mlr.press/v80/huang18d.html (PMLR, 2018).

[35] Jaini, P., Selby, K. A. & Yu, Y. Sum of squares polynomial flow. Preprint at *arXiv* http://arxiv.org/abs/1905.02325 (2019).

