# Supplementary material for: Parameterized quantum circuits as universal generative models for continuous multivariate distributions
Source: npj Quantum Inf. 2025 Jul 22;11(1):121. doi: 10.1038/s41534-025-01064-3 (PMC12283343; doi:10.1038/s41534-025-01064-3)
Supplement: Supplementary file 1 — Supplementary Information pdf [file 41534_2025_1064_MOESM1_ESM.pdf]

Supplemental Information for "Parameterized quantum circuits as universal generative models for continuous multivariate distributions"

I. UNIVERSALITY DEFINITION

In this appendix, we spend a bit more time justifying our choice of the definition of universality and relating it to concepts of interest. The definition of universality for generative models hinges on the notion of closeness between two random variables. In the case of generative modelling, *convergence in distribution*, a common concept in probability theory, is often sought. For example, the central limit theorem precisely states the average of any L independent random variables with mean μ and variance Σ converges in distribution to the normal distribution $\mathcal{N}(\mu, \Sigma/L)$.

Definition 1 (Universal generative model family)**.** *A generative model is a family of parameterized sampling procedures that enable the sampling from a corresponding set of M-dimensional probability density functions $\mathcal{P}(\mathcal{X})$ on $\mathcal{X} \subseteq \mathbb{R}^M$.*

A generative model is called universal if for every probability density function q on $\mathcal{X}$ there exists a sequence $\{p_k | p_k \in \mathcal{P}(\mathcal{X})\}_{1\leq k \leq \infty}$ such that the sequence of random variables $X_k \sim p_k$ converges in distribution to $X \sim q$. Equivalently, this means that the sequence of cumulative distribution functions of p_k, which we call P_k, converges pointwise to the cumulative distribution function of q, which we call Q.

$$\forall x \in \mathcal{X}, \lim_{k\to\infty} P_k(x) = Q(x). \tag{1}$$

In this work, we will mostly consider distributions with finite support, because this is the case in most practical real-world problems. In particular, their probability density functions are integrable functions and convergence in distribution implies convergence in the first Wasserstein distance W_1 [1]. For completeness, we recall below the definition of the Wasserstein distance, also known as the Earth Mover's Distance, that we use in the context of this work.

Definition 2 (Wasserstein distance)**.** *The k-th Wasserstein distance between two probability density functions p and q on $[-1,1]^M$ is defined as:*

$$W_k(p,q) = \left(\inf_{\gamma\in\Pi(p,q)} \int_{\mathbb{R}^2} \|x-y\|^k d\gamma(x,y) \right)^{\frac{1}{k}}, \tag{2}$$

where $\Pi(p,q)$ is the set of couplings of p and q, and $\|\cdot\|$ denotes the Euclidean distance. The parameter $k \geq 1$ determines the so-called order of the Wasserstein distance.

For the rest of the paper, we will only consider the first-order Wasserstein distance ($k = 1$) and therefore simply refer to it as the Wasserstein distance.

Importantly, universality is defined on a given support noted as $\mathcal{X}$ in Definition 1. For this work, we choose the support to be the hypercube $\mathcal{X} = [-1,1]^M$, because the first step of most machine learning pipelines is to rescale the data to fit on a given interval. Note that the length of the hypercube can be rescaled, by rescaling the norm of the observables. Finally, since any distribution with infinite support but finite moments can be arbitrarily approximated by a distribution with finite support, this means that if we allow the observable norm to scale, we can also approximate any distribution with finite moments (but perhaps infinite support). We make this explicit below.

We consider a distribution with probability density function p_Y with infinite support $\mathbb{R}^M$ and finite moments. The goal is to find a sequence of random variables with bounded support probability density function that converges pointwise to p_y. We define the sequence of random variables $\{Y_k\}_{1\leq k\leq\infty}$ whose probability density functions p_{Y_k} are proportional to that of Y on the hypercube $[-k-k_0, +k+k_0]^M$, where k_0 is the first integer such that Y has non-null support on the hypercube. This sequence converges in distribution to Y.

II. UNIVERSALITY PROOFS

In this appendix, we provide the proofs for Theorems 1 and 2 that state the universality of two families of EVS. First, we provide more details on random variable mapping which is the core concept of the proof in Section II A.

For both proofs, we use Theorem 1 from [2]. For the first theorem, in Section II B we modify universality results from functions as coordinates in the computational basis to functions as the expectation value of an observable. In Section II C we broaden the universality of quantum reuploading models to some discontinuous functions by relaxing the required strength of convergence. More precisely we go from the uniform density in bounded continuous functions to the pointwise density in bounded piece-wise continuous functions. Finally, by stacking M universal circuits, we extend universality to multivariate output functions. All these extensions of [2] together yield Theorem 4. With this theorem, we show that the *product encoding* circuit satisfies the universal mapping property, and therefore, using the concept of random variable mapping, yields a universal generative model. For the *observable dense encoding* in Section II D we follow a similar strategy, but instead, each output variable is encoded as the overlap between the output state and each computational basis state. With the normalisation of the quantum states the observables are projectors on computational basis states, amplified by a factor proportional to the dimension of the target distribution.

A. Constructive mapping between a random variable and the uniform distribution

Let us consider an absolutely continuous random variable Y with probability density function p_Y on a bounded set $[a,b]^M$. We recall a definition of an absolutely continuous variable below.

Definition 3. *A random variable X is said to be absolutely continuous if its cumulative distribution function (CDF) can be expressed as the integral of a non-negative function, known as the probability density function (PDF).*

This excludes, for example, Dirac deltas. In what follows, we construct an invertible mapping to transform the uniform random variable X into this random variable $Y = [Y_k]$. We call $G_1 : [0,1] \to [a,b]$ the cumulative distribution function of the marginal of Y_1. It is invertible and we define F_1 as its inverse,

$$Y_1 = F_1(X_1), X_1 = G_1(Y_1). \tag{3}$$

Next we consider the marginal of Y_2 conditioned by Y_1, we define the cumulative distribution $G_2 : [a,b]^2 \to [0,1]$,

$$G_2(y_1, y_2) = P(Y_2 = y_2 | Y_1 = y_1). \tag{4}$$

It is invertible with respect to Y_2,

$$G_2^{-1}(Y_1, X_2) = Y_2 \iff G_2(Y_1, Y_2) = X_2. \tag{5}$$

We define $F_2 : [0,1]^2 \to [a,b]$ as follows,

$$F_2(X_1, X_2) = G_2^{-1}(F_1(X_1), X_2). \tag{6}$$

We have $Y_2 = F_2(X_1, X_2)$. Continuing this process iteratively for all coordinates, it is possible to fully define the invertible mapping $F = [F_k]$ with inverse $G = [G_k]$ such that $Y = F(X)$. This is the essence of triangular mapping in [3]. In addition, this mapping is bounded and piece-wise continuous, because it is composed of inverse cumulative distribution functions of absolutely continuous variables on bounded support.

B. From state coordinate universality to expectation of observable universality

We start by recalling Theorem 4 from [2], which proves that the following circuit is universal. It has L layers, $\theta \in \mathbb{R}^{(M+2)\times L}$ parameters and for $x \in \mathbb{R}^M$ is defined as

$$U_\theta(x) \coloneqq \prod_{l=1}^{L} R_y(\theta_{0,l}) \left(\prod_{m=1}^{M} R_z(x_m \theta_{m,l}) \right) R_z(\theta_{M+1,l}). \tag{7}$$

Theorem 1 (from [2]). *For any pair of functions and real number*

$$(f \in \mathcal{C}([0,1]^M \to [0,1)), \phi \in \mathcal{C}([0,1]^M \to [0,2\pi)), \epsilon > 0)$$

There exists a one qubit circuit $U : [0,1]^M \to \mathcal{U}(2)$ s.t.

$$\forall x, \left| \langle 1 | U(x) | 0 \rangle - f(x) e^{i\phi(x)} \right| < \epsilon. \tag{8}$$

In this work, $\mathcal{C}$ is the set of continuous functions. This theorem yields the universality of functions embedded in a quantum state in the uniform sense. In the context of expectation value sampling, we are interested in the universality of function as the expectation value of a unit norm observable, captured by the following theorem.

Theorem 2. *For any function $g \in \mathcal{C}([0,1]^M \to [-1,1])$ and for any $\epsilon > 0$, there exists a one qubit circuit $U(x) : [0,1]^M \to \mathcal{U}(2)$ and an observable O with unit spectral norm $\|O\| = 1$ s.t.*

$$\forall x, \left|\langle 0| U^\dagger(x) O U(x) |0\rangle - g(x)\right| < \epsilon. \tag{9}$$

Proof: We are given an arbitrary function $g \in \mathcal{C}([0,1]^M \to [-1,1])$ and $\epsilon > 0$. We define the function $f = \sqrt{\frac{g+1}{2}}$ which is well defined on $\mathcal{C}([0,1]^M \to [0,1])$. We apply Theorem 1 to $(f, \phi = 0, \epsilon/4)$ and get a circuit U that yields a state close to

$$|x\rangle = \sqrt{1 - f(x)^2}\, |0\rangle + f(x) |1\rangle. \tag{10}$$

The Z expectation value of the above state is

$$\langle x| Z |x\rangle = 2f(x)^2 - 1 = g(x). \tag{11}$$

We are now going to prove that the expectation value is close to the target function g. First, we note that the square function is 2-Lipschitz on $[0,1]$ and therefore for every pair of real numbers $(x,y) \in [0,1]^2$

$$|x - y| < \epsilon \implies \left|x^2 - y^2\right| < 2\epsilon. \tag{12}$$

We define $p_{0/1}$ the probabilities of measuring $U(x)|0\rangle$ in state $|0\rangle$ and $|1\rangle$ respectively. Recalling that

$$|\langle 1| U(x) |0\rangle - f(x)| < \epsilon/4, \tag{13}$$

we can write

$$\left|p_1 - f(x)^2\right| < \epsilon/2 \tag{14}$$

$$\left|p_0 - (1 - f(x)^2)\right| = \left|1 - |\langle 1| U(x) |0\rangle|^2 - (1 - f(x)^2)\right| < \epsilon/2. \tag{15}$$

Finally,

$$|\langle Z\rangle - g(x)| \leq \left|p_1 - f(x)^2\right| + \left|p_0 - (1 - f(x)^2)\right| < \epsilon. \tag{16}$$

This yields the uniform density of quantum functions in the set of bounded continuous functions.

C. From uniform density on continuous functions to pointwise density on discontinuous functions

We first start by highlighting the difference between uniform convergence and pointwise convergence and illustrate it with an example.

Definition 4 (Pointwise convergence)**.** *Let $\{f_k | f_k : \mathcal{X} \to \mathbb{R}\}_{1 \leq k \leq \infty}$ be a sequence of functions, and let $f : \mathcal{X} \to \mathbb{R}$ be another function defined on the same domain $\mathcal{X}$. We say that the sequence $\{f_k\}$ converges pointwise to f if, for each $x \in \mathcal{X}$, the sequence of real numbers $\{f_k(x)\}_{1 \leq k \leq \infty}$ converges to $f(x)$ as k approaches infinity,*

$$\lim_{n \to \infty} f_k(x) = f(x) \quad \text{for all } x \in \mathcal{X}.$$

Definition 5 (Uniform convergence)**.** *Let $\{f_k | f_k : \mathcal{X} \to \mathbb{R}\}_{1 \leq k \leq \infty}$ be a sequence of functions, and let $f : \mathcal{X} \to \mathbb{R}$ be another function defined on the same domain $\mathcal{X}$. We say that the sequence f_k converges uniformly to f if, for any given $\epsilon > 0$, there exists an $K \in \mathbb{N}$ such that for all $k \geq K$ and for all $x \in \mathcal{X}$, the difference $|f_k(x) - f(x)|$ is less than ϵ,*

$$\forall \epsilon > 0, \exists K \in \mathbb{N} : \forall k \geq K, \forall x \in \mathcal{X}, |f_k(x) - f(x)| < \epsilon.$$

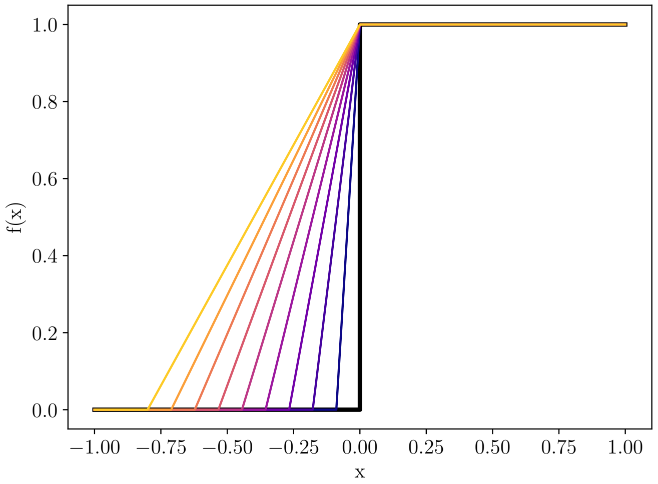

FIG. 1. A sequence of continuous functions converging pointwise but not uniformly to the step function.

Uniform convergence is stronger, it implies pointwise convergence, but the reverse is not true. For example, consider the step function f.

$$f(x) = \begin{cases} -1, & \text{if } x \in [-1, 0[\\ +1, & \text{if } x \in [0, +1] \end{cases} \tag{17}$$

It is impossible to define a sequence of continuous functions that would uniformly converge to it, however, it is possible to have a sequence of continuous functions that converges pointwise to it, see Figure 1.

$$f_k(x) = \begin{cases} -1, & \text{if } x \in [-1, 1/k[\\ 1 + kx, & \text{if } x \in [-1/k, 0] \\ +1, & \text{if } x \in]0, +1] \end{cases} \tag{18}$$

In [4] Baire defined hierarchical pointwise convergence classes of functions. Baire class 0 is the set of continuous functions, and the class c is the set of functions that are the pointwise limit of class $c - 1$. In particular, in [5, 6], it was proven that bounded piece-wise continuous functions are of class 1. This means that for any bounded piece-wise continuous function f, there exists a sequence of bounded continuous functions that converges pointwise to f. This means that bounded continuous functions are dense in bounded piece-wise continuous functions in the pointwise topology. Therefore, building on Theorem 2, we have the following theorem, writing $\mathcal{B}$ as the set of piecewise continuous functions.

Theorem 3. *For any function $f : \mathcal{B}([0,1]^M \to [-1,1])$, there exists a sequence (indexed by k) of one qubit circuits and observables with unit spectral norm,*

$$\{(U_k(x) : [0,1]^M \to \mathcal{U}(2), O_k)\}_{1 \leq k \leq \infty} \tag{19}$$

such that the sequence of functions $\{g_k\}$ with

$$g_k(x) = \langle 0| U_k^\dagger(x) O_k U_k(x) |0\rangle \tag{20}$$

converges pointwise to f.

D. Construction of the observable dense encoding circuit

Note: We have used slight abuse of notations in the below proof to increase readability, specifically in the approximations noted as $\equiv_\epsilon$.

Considering architecture such as in [7] and universal one qubit gate as $R_x(\alpha)R_z(\beta)R_x(\gamma)$, for any number of qubits, it is possible to design a circuit $U : [0, 2\pi)^L \to \mathcal{U}(2^n)$ made only of a finite number of fixed gates (CNOT and constant rotations) and parameterized σ_z rotations gates that can reach any pure quantum state when applied to state $|0\rangle$,

$$\forall |\psi\rangle, \exists \theta \in [0, 2\pi)^L, |\psi\rangle = U(\theta) |0\rangle . \tag{21}$$

Let's consider a distribution p_ψ over pure states. Because the architecture above can reach any pure state, there exists a corresponding distribution over the parameters p_θ such that the distribution $V(\theta) |0\rangle, \theta \sim p_\theta$ matches perfectly p_ψ in distribution. We define $g : [0, 1]^L \to [0, 2\pi)^L$ as the mapping that transforms the uniform distribution over $[0, 1]^L$ into p_θ. We have $V(f(X)) |0\rangle, X \sim U([0, 1]^L)$ matches perfectly p_ψ in distribution, where V is composed of a finite number of fixed gates and L σ_z rotation gates parameterized by $g_l(X)$.

Lemma 1. *For any distribution over pure states p_ψ, there exists a circuit architecture V made of constant gates and parameterized σ_z rotations, and a mapping g such that*

$$X \sim U([0, 1]^L), V(g(X)) |0\rangle \sim p_\psi \tag{22}$$

Next, we decompose $R_z \circ g$ gates into a sequence of constant gates and parameterized σ_z rotations.

From [2], in the proof in the appendix, it is shown that there exists a quantum circuit W taking a multidimensional input and approximating the following parameterized quantum gate,

$$\forall f : [0, 1]^L \to [0, 1], \phi : [0, 1]^L \to [0, 2\pi), \exists W, W(x) \equiv_\epsilon \begin{bmatrix} \sqrt{1 - f(x)^2} e^{+i\phi(x)} & -f(x)e^{+i\phi(x)} \\ f(x)e^{-i\phi(x)} & \sqrt{1 - f(x)^2} e^{-i\phi(x)} \end{bmatrix} . \tag{23}$$

Choosing $\phi = 0$, and $f = \sin g$, we have $\sqrt{1 - f^2} = \cos g$, and we note that $W(x) = R_z \circ g$. We can conclude the following.

Lemma 2.

$$\forall f : [0, 1]^L \to [0, 1], \exists W, R_z \circ f \equiv_\epsilon W, \tag{24}$$

where W is a quantum circuit made of constant gates and R_z gates applied to individual components of x.

Combining both above lemmas, we get the following.

Lemma 3. *For any distribution over pure states p_ψ, there exists a circuit architecture V made of constant gates and parameterized z rotations such that*

$$X \sim U([0, 1]^L), V(X) |0\rangle \sim_\epsilon p_\psi . \tag{25}$$

We are now going to use that lemma to prove that n-qubits expectation value samplers are universal for $\exp(n)$-dimensional distributions with constant support if the observables are allowed to have $\exp(n)$ norms.

We are given an arbitrary random variable Y following a M-dimensional distribution p_Y with support $[-1, 1]^M$. We define the following state over n qubits with $M = 2^n - 1$.

$$|\psi(Y)\rangle = \sum_{m \le M} Z_m |m\rangle + \sqrt{1 - \sum_{m \le M} Z_m^2} \, |M + 1\rangle \tag{26}$$

$$Z_m = \sqrt{\frac{Y_m + 1}{2M}} \tag{27}$$

We define as p_ψ as the probability density functions over states when $Y \sim p_Y$, using the previous lemma we get a circuit W composed only of constant gates and z rotations gates with one of the L parameters as input that approximates p_ψ. We define the observables $\forall m \le M, O_m = 2M |m\rangle \langle m| - I$. They have spectral norm $\|O_m\| = 2M - 1 = \Theta(2^n)$. In addition, $\langle \psi(Y)| O_m |\psi(Y)\rangle = Y_m$. Therefore, the expectation value sampler $(W, O, U([0, 1]^L))$ approximates p_Y. This concludes the proof to Theorem 2.

III. PROOF OF NECESSARY RESOURCES FOR UNIVERSALITY

In this appendix, we prove Theorem 3 about the necessary resources for EVS to achieve universality, which we recall below.

Theorem. *For an n-qubit expectation value sampling model $(U_\theta, \mathbf{O}, p_X)$ to be able to approximate any distribution with support in $[-1,1]^M$ to any accuracy $\epsilon > 0$ with respect to the Wasserstein distance, it is necessary that for every $m \leq M$:*

1. $\lambda_{\min}(O_m) \leq -1+\epsilon$ *and* $\lambda_{\max}(O_m) \geq +1-\epsilon$
2. $n \in \Omega\left(\frac{M(1-\epsilon)^2}{\Lambda(O_m)}\right) \subseteq \Omega\left(\frac{M(1-\epsilon)^2}{\|O_m\|^2}\right)$

with $\lambda_{\min/\max}(O)$ returning respectively the minimum and maximum eigenvalues of observable O, and $\Lambda(O) := -\lambda_{\min}(O)\lambda_{\max}(O)$.

Proof: Let's suppose there is an n-qubit expectation value scheme with M observables $\mathbf{O}$ that is able to approximate any distributions with support included in $[-1,1]^M$ to ϵ with respect to the first Wasserstein distance W_1.

Because the expectation value model is universal, it means that for any vertex of the hypercube $c \in \{-1,1\}^M$, it can approximate the Dirac delta at c. We then use the lemma below.

Lemma 4. *Any distribution that is ϵ-close (in the Wasserstein distance) to the Dirac delta at a given point must have nonzero support within the Euclidean distance sphere centred in that point with radius ϵ.*

This means that there is non-zero support on the ϵ sphere around c, and therefore there exists a quantum state ρ_c whose list of expectations is ϵ-close to that point. This yields the following result.

$$\forall c \in \{-1,1\}^M, \exists \rho_c, \sum_{m=1}^{M} (\mathrm{Tr}(O_m\rho_c) - c_m)^2 \leq \epsilon^2. \tag{28}$$

Because the above is a sum of positive components, we can write $\forall m$

(a) if $c_m = 0$, then $-1-\epsilon \leq \mathrm{Tr}(O_m\rho_c) \leq -1+\epsilon$

(b) if $c_m = 1$, then $+1-\epsilon \leq \mathrm{Tr}(O_m\rho_c) \leq +1+\epsilon$

The above yields conditions on the spectrum of O_m. We note $\lambda_{\min}$ and $\lambda_{\max}$, respectively the minimum and maximum eigenvalues of O_m. We define $\gamma := 1-\epsilon$. We know that $\forall \rho, \mathrm{Tr}(O\rho) \geq \lambda_{\min}$ therefore, $-\gamma \geq \lambda_{\min}$, the same reasoning applies for the maximum eigenvalue, yielding:

(a) $\lambda_{\min} \leq -\gamma$,

(b) $\lambda_{\max} \geq +\gamma$.

For the rest of the proof, we combine approaches from the proof of theorem 2.6 in [8] and that of B.1 in [9]. We define the two-outcome POVMs $\{E_m, I-E_m\}$ with

$$E_m = \frac{O_m - \lambda_{\min}}{\lambda_{\max} - \lambda_{\min}} \tag{29}$$

We define $\beta := \frac{-\lambda_{\min}}{\lambda_{\max}-\lambda_{\min}}$. The spectral inequalities yield $\beta \geq \frac{1}{2}$. With this definition, the two conditions above translate to:

(a) $c_m = 0, p_0 := \mathrm{Tr}(E_m\rho_c) \leq \beta - \frac{\gamma}{\lambda_{\max}-\lambda_{\min}}$

(b) $c_m = 1, p_1 := \mathrm{Tr}(E_m\rho_c) \geq \beta + \frac{\gamma}{\lambda_{\max}-\lambda_{\min}}$

We define the amplified Positive Operator-Valued Measures (POVMs) that apply $\{E_m, I-E_m\}$ to $L \geq 1$ copies of ρ and return 1 if and only if at least βL copies of the original POVMs return 1.

From Holevo's bound (found as Theorem 5.1 in [10]), for the amplified scheme to correctly identify the corresponding bit $b_m = (c_m+1)/2$ with probability q it is necessary that

$$nL \geq (1-H(q))M, \tag{30}$$

where H is the binary entropy function.

We define the random variable $X_{m,l}$ which takes the value of the output of the POVM of the m-th observable on the l-th copy. We define $X_m^{(L)} := \frac{1}{L}\sum_l X_{m,l}$.

In the case $c_m = -1$, we have $\mathbb{E}[X_m^{(L)}] = p_0$. The probability of the amplified POVMs yielding the wrong output is

$$P(X_m^{(L)} > \beta) \leq P\left(X_m^{(L)} > p_0 + \frac{\gamma}{\lambda_{\max} - \lambda_{\min}}\right) \tag{31}$$

Recalling that $\beta \geq 1/2$, we can use the Chernoff bound on the Bernoulli variable $X_m^{(L)}$ and we get

$$P(X_m^{(L)} > p_0 + \frac{\gamma}{\lambda_{\max} - \lambda_{\min}}) \leq \exp\left\{-\frac{\gamma^2 L}{2(-\lambda_{\min})\lambda_{\max}}\right\} \tag{32}$$

We define $\Lambda = (-\lambda_{\min})\lambda_{\max}$, we have $\gamma^2 \leq \Lambda \leq \|O\|^2$.

For the probability of the amplified POVMs to yield the correct output with probability q it is necessary that $P(X_m^{(L)} \leq \beta) \geq q$.

Finally, we get

$$\log(1/q) \leq \frac{\gamma^2 L}{2\Lambda} \tag{33}$$

Combining Chernoff's and Holevo's inequalities, we conclude the proof of Theorem 3:

$$\Lambda \geq \frac{1 - H(q)}{\log(1/q)} \frac{\gamma^2 M}{n}. \tag{34}$$

Note: The above is a tighter condition than in Theorem 2.6 in [9] but falls back to it, when $\Lambda = \|O\|^2$, which corresponds to $\lambda_{\min} = -\|O\|$ and $\lambda_{\max} = \|O\|$. In the opposite scenario, we have $\Lambda = \gamma^2$, which corresponds to constant norm observables, yielding $n \in \Omega(M)$. The norm of observables affects the number of measurements to reach a desired additive accuracy.

Note: The above necessary conditions use results which involve a more general case where we assume that prior to measurement, we use a general parameterized quantum channel which can also prepare mixed states. However, we can reduce this to the special case of unitaries as well. By purification, mixed states can be mimicked by using $2n$ qubits pure states, since we are only interested in scalings, the factor 2 plays no role in the second condition of Theorem 3, and the necessary conditions also hold for pure states.

IV. APPROXIMATION OF EXPECTATION VALUES

In this appendix, we justify the connection between the spectral norm of observables and the number of measurements. In practice, we do not have access to exact expectation values and we have to estimate them through sampling. This creates a distribution $p_{\hat{Y}}$ (n dimensional) slightly different from the distribution with exact expectation values p_Y. For simplicity, we assume that shot noise $\rho_\epsilon \sim \mathcal{N}(0, \epsilon I_n)$ (n dimensional) acts like an additional Gaussian noise with zero mean and standard deviation ϵ. ρ_ϵ and Y are independent and we consider the random variable $\hat{Y} = Y + \rho_\epsilon$. Therefore the density $p_{\hat{Y}}$ is the convolution of p_Y and p_{ρ_ϵ}. Using Lemma 7.1.10 from [11], we know that the Wasserstein distance between p_Y and $p_{\hat{Y}}$, $W_p(p_Y, p_{\hat{Y}}) \in O(\epsilon)$.

Lemma 5. *An arbitrary expectation value sampler outputs an M-dimensional random vector $Y \sim p_Y$ with an infinite number of measurements, i.e. with access to exact expectation values. We consider the same circuit but with a finite number of measurements T that estimates expectation value by sampling and averaging for each observable and yields a random vector $\hat{Y} \sim p_{\hat{Y}}$. The number of measurements T required to guarantee that the Wasserstein distance between both distributions is smaller than ϵ satisfies*

$$T \in \Theta\left(\frac{M\|O\|}{\epsilon^2}\right). \tag{35}$$

In practice, different techniques exist to estimate expectation values of observables with different degrees of measurement efficiency, with shadow tomography techniques [12] surpassing the "vanilla estimation". For simplicity, we consider a vanilla estimation where each observable with norm $\|O\|$ is measured t times and the average is returned.

This yields a shot noise close to the Gaussian model above with $\epsilon^2 \in \Theta(\|O\|/t)$. The total number of measurements T is then $T = tM$, which yield $T \in \Theta(M\|O\|/\epsilon^2)$

Note that the first result cannot be trivially applied to techniques such as shadow tomography [12]. Indeed, the corresponding shot noise ρ cannot, in general, be modelled by a Gaussian independent noise, or at least the covariance matrix should not be proportional to the identity.

V. ADDITIONAL EXPRESSIVITY TOOLS

In this appendix, we provide additional tools to analyze the expressivity of expectation value samplers. Specifically, the choice of observables, and the choice of random variable encoding.

A. Primary mapping and the choice of observables

We are using the standard Pauli basis for the space of $2^n \times 2^n$ Hermitian operators $\mathbf{P}$. It is composed of all possible combinations of n Pauli matrices $\sigma_{0,1,2,3}$, which yields $|\mathbf{P}| = 4^n$. We formalize as follows

$$\mathbf{P} := (P_k, k \in \{0,1,2,3\}^n) \tag{36}$$
$$= (\otimes_{1\leq i\leq n}\sigma_{k_i}, k_i \in \{0,1,2,3\}). \tag{37}$$

Any vector of M observables $\mathbf{O} = (O_m)$, can be expressed as a linear mapping applied on the vector of all Pauli strings: $\mathbf{O} = A\mathbf{P}$, where A is an $M \times 4^n$ matrix, and $\mathbf{P}$ is a 4^n dimensional vector. Therefore the distribution associated with the Pauli basis encompasses any distributions, which leads us to define the primary mapping as follows.

Definition 6 (Primary mapping). *The primary mapping g of an n-qubit encoding circuit $U_\theta(x)$ is defined as the mapping of the associated expectation value sampling model with the Pauli basis $\mathbf{P}$, defined as $(U_\theta(x), \mathbf{P}, p_X)$ according to the definition in the main body. It can be expressed as follows*

$$x \in [0, 2\pi)^N \xrightarrow{g} \left(\langle 0| U_\theta(x)^\dagger P_k U_\theta(x) |0\rangle\right)_{1\leq k\leq 2^n}. \tag{38}$$

It yields the 4^n-dimensional random variable $Z = g(X)$ when $X \sim p_X$.

It is easy to see that any distribution obtained by an expectation value sampling model can always be expressed by considering an intermediary output of the 4^n set of observables, followed by the linear mapping A. We capture this idea in the following theorem.

Lemma 6. *Given an encoding circuit $U_\theta(x)$ and random variable with distribution p_X, for any the choice of observables $\mathbf{O}$, the expectation value sampling model $(U_\theta(x), \mathbf{O}, p_X)$ is a linear transformation of the expectation value sampling model $(U_\theta(x), \mathbf{P}, p_X)$, where $\mathbf{P}$ is the Pauli basis per definition of the primary mapping in Definition 6.*

This concept of primary mapping has immediate consequences on the possible correlation of output variables of expectation value sampling models. For a given data encoding part on n qubits, the primary mapping will yield a random variable Z with a covariance matrix C_z. Because any expectation value sampling model based on the same data encoding is a linear transformation of the primary mapping, the number of uncorrelated variables is limited by the number of non-null eigenvalues of the covariance matrix C_z, which is upper bounded in any case by $4^n - 1$.

Lemma 7. *Given an encoding circuit $U(x)$ over n qubits and a random variable with distribution p_X, we note L the number of non-zero eigenvalues of the covariance matrix of the primary mapping. For any choice of observables, any expectation value model using $(U(x), p_X)$ will yield at most L uncorrelated variables. In addition, $L \leq 4^n - 1$.*

B. Random variable encoding as a polynomial chaos expansion

After focusing on the choice of observables, in this subsection, we analyze the impact of the choice of the input random variable and the circuit encoding on the expressivity. We propose the polynomial chaos expansion [13] as a useful tool to analyze the expressivity of expectation value sampling models, as the analogue of the Fourier decomposition. The general polynomial chaos expansion is a representation of random variables as a vector in a Hilbert space of orthogonal functions, as defined below.

Normal distribution	Hermite polynomials
Uniform distribution	Legendre polynomials
Exponential distribution	Laguerre polynomials
Beta distribution	Jacobi polynomials

TABLE I. Pairs of distributions and corresponding orthonormal families commonly used in General Polynomial Chaos expansion.

Definition 7 (Generalized Polynomial Chaos Expansion)**.** *A generalized chaos expansion is characterized by a probability density function p_X defined on the support $\mathcal{X} \subseteq \mathbb{R}^M$ with finite moments (usually chosen as standard distributions, such as Gaussian or uniform). This choice defines an inner product for functions in $\{f : \mathcal{X} \to \mathbb{R}\}$:*

$$\langle f|g\rangle_{p_X} := \int_{\mathcal{X}} f^*(x)g(x)p_X(x)dx. \tag{39}$$

This choice of inner product comes with the choice of an ordered family of functions, usually polynomials, that are orthonormal with respect to the above inner product.

$$\Phi_{p_X} = \{\phi_l : \mathcal{X} \to \mathbb{R}, \forall(k,l), |\phi_l\rangle \langle\phi_k| = \delta_{k,l}\} \tag{40}$$

A generalized chaos expansion is a representation of a random variable Y with probability density p_Y as a vector α in this Hilbert space, such that:

$$Y = \sum_{l=0}^{\infty} \alpha_l \phi_l(X) \sim p_Y, X \sim p_X \tag{41}$$

This provides a Hilbert space as a potential structure to study random variable mappings. In particular, one of the main results of polynomial chaos expansion is that they are universal generative models.

Common pairs of distribution and associated orthogonal polynomials family can be found in Table I. In the context of expectation value sampling, we focus on the family of functions orthonormal with respect to the inner product associated with the uniform distribution on $\mathcal{X} = [0, 2\pi)^M$

$$\Phi = \{ \prod_{1\leq m\leq M} e^{ik_m x_m}, k \in \mathbb{Z}^M\} \tag{42}$$

Quantum reuploading circuits are a widely used class of parameterized quantum circuits that output a function of the data. They are used in a regressive context, where optimization techniques are used such that their output fits a target function. It has been widely studied and used that if they use integer-valued spectrum Hamiltonian, their output hypothesis function can be decomposed as an exact finite Fourier series [14]. This means that there exists $c_{\mathbf{k},l} \in \mathbb{C}$ such that the hypothesis function f can be exactly written as a finite Fourier series:

$$f(x) = \sum_{k_0,k_1,\cdots,k_M=-K}^{+K} c_{\mathbf{k},l} \prod_{1\leq m\leq M} e^{ik_m x_m},. \tag{43}$$

This fact extends to a generative modelling context where expectation value sampling models using integer-valued quantum reuploading circuits yield distributions with an exact finite polynomial chaos expansion. We formalize this below.

Theorem 4. *Any expectation value sampling model using a quantum reuploading model with integer-valued spectrum $U_\theta(x)$, together with the uniform distribution on $[0, 2\pi)^M$ outputs a random variable Y that has an exact finite polynomial chaos expansion for any choice of observables.*

This subsection formalizes the tight connection between quantum circuits used in a regressive context and their use in a generative context. Therefore, it is expected that, beyond the universality, many properties of such models can be transferred from a regressive context to a generative context, but we leave that for future work.

C. Choice of input distribution

Lastly, we discuss the choice of input random variable, noted as p_X. We highlight that the universality theorems in this work apply to the uniform distribution. This, together with generalized chaos expansion considerations, makes the uniform distribution a natural choice for the input random variable of expectation value samplers. This is due mostly to the fact that it has a bounded support. Indeed, it is known that the most commonly used parameterized quantum circuits are periodic, in particular when they have a finite Fourier decomposition. This is also why the universality of quantum reuploading circuits is proven for functions on bounded domains, corresponding to a half period. In contrast, let us consider an expectation value sampler $(U, \mathbf{O}, p_X)$, where the input random variable follows a Gaussian distribution $X \sim \mathcal{N}(0, 1)$, which has unbounded support and for which the mapping f is 1-periodic. $X = 0$ is the highest probability event and will yield the same output as a very low probability event, for example, $X = 100$. This means that a very low probability input event and a very high probability input event will yield the same output sample, which is a feature rarely considered desirable. This choice of uniform distribution is in contrast to classical GANs where Gaussian distributions are typically preferred.

---

[1] Cédric Villani. *Optimal Transport*, volume 338 of *Grundlehren der mathematischen Wissenschaften.* Springer, Berlin, Heidelberg, 2009.

[2] Adrián Pérez-Salinas, David López-Núñez, Artur García-Sáez, P. Forn-Díaz, and José I. Latorre. One qubit as a Universal Approximant. *Physical Review A*, 104(1):012405, July 2021. arXiv:2102.04032 [quant-ph].

[3] V. I. Bogachev, A. V. Kolesnikov, and K. V. Medvedev. Triangular transformations of measures. *Sbornik: Mathematics*, 196(3):309, April 2005. Publisher: IOP Publishing.

[4] R. Baire. Sur les fonctions de variables réelles. *Annali di Matematica Pura ed Applicata (1898-1922)*, 3(1):1–123, December 1899.

[5] M. Laczkovich. Baire 1 functions. *Real Analysis Exchange*, 9(1):15–28, January 1984. Publisher: Michigan State University Press.

[6] H. Lebesgue. Une propriété caractéristique des fonctions de classe 1. *Bulletin de la Société Mathématique de France*, 32:229–242, 1904. Publisher: Société mathématique de France.

[7] Martin Plesch and Časlav Brukner. Quantum-state preparation with universal gate decompositions. *Physical Review A*, 83(3):032302, March 2011. Publisher: American Physical Society.

[8] Scott Aaronson. The learnability of quantum states. *Proceedings of the Royal Society A: Mathematical, Physical and Engineering Sciences*, 463(2088):3089–3114, September 2007. Publisher: Royal Society.

[9] Sofiene Jerbi, Casper Gyurik, Simon C. Marshall, Riccardo Molteni, and Vedran Dunjko. Shadows of quantum machine learning, May 2023. arXiv:2306.00061 [quant-ph, stat].

[10] Andris Ambainis, Ashwin Nayak, Amnon Ta-Shma, and Umesh Vazirani. Dense quantum coding and quantum finite automata. *Journal of the ACM*, 49(4):496–511, July 2002.

[11] Luigi Ambrosio, Nicola Gigli, and Giuseppe Savaré. *Gradient Flows.* Lectures in Mathematics ETH Zürich. Birkhäuser-Verlag, Basel, 2005.

[12] Kevin Chen, Andrii Kurkin, Hao Wang, and Vedran Dunjko. in preparation.

[13] Dongbin Xiu. *Numerical Methods for Stochastic Computations: A Spectral Method Approach.* Princeton University Press, 2010.

[14] Maria Schuld, Ryan Sweke, and Johannes Jakob Meyer. Effect of data encoding on the expressive power of variational quantum-machine-learning models. *Physical Review A*, 103(3):032430, March 2021. Publisher: American Physical Society.
